# Does Chronic Kidney Disease Result in High Risk of Atrial Fibrillation?

**DOI:** 10.3389/fcvm.2019.00082

**Published:** 2019-06-20

**Authors:** Dapeng Zhang, Yibin Feng, Feona Chung-Yin Leung, Lingchong Wang, Zhimin Zhang

**Affiliations:** ^1^Department of Chinese Medicine, The First Affiliated Hospital of Guangzhou Medical University, Guangzhou, China; ^2^Li Ka Shing (LKS) Faculty of Medicine, School of Chinese Medicine, The University of Hong Kong, Hong Kong, China; ^3^School of Pharmacy, Nanjing University of Chinese Medicine, Nanjing, China

**Keywords:** chronic kidney disease, atrial fibrillation, hypertension, renin–angiotensin–aldosterone system, atrial structural remodeling

Chronic kidney disease (CKD), defined as either pathological albuminuria (>30 mg/24 h) or decreased GFR (<60 mL/min/1.73 m^2^), is associated with an increase in cardiovascular morbidity and mortality (1, 2). Atrial fibrillation (AF), the most common sustained arrhythmia associated with increased risk of ischaemic and haemorrhagic stroke, is increasingly prevalent with the decline of kidney function (3). It was reported that the occurrence risk of AF began to grow as renal dysfunction deteriorated (CKD at stage 3: HR = 1.6, 95% CI = 1.27–2.13; at stage 4: HR = 3.2, 95% CI = 2.0–5.0) (4). Similar results from a Medicare cohort pointed out that CKD increases the 2-year incidence of AF to 12.2% at stages 1 and 2, and to 14.4% at stages 3–5, compared with 7.5% for patients without CKD (5). Although the incidence of AF in hypertensive patients with CKD is significantly increased (adjusted HR = 2.18, 95% CI: 1.2–3.9), it could be independently increased in advanced CKD (stage 4/5) patients (6). On the contrary, the improvement of kidney function could help reduce the recurrence of AF in patients undergoing catheter ablation for AF (7).

CKD and AF share several common predisposing factors including hypertension, heart failure, coronary artery disease, obesity, and diabetes, etc (8). CKD activates the renin–angiotensin–aldosterone system (RAAS) and sympathetic nervous system and induces oxidative stress, systemic inflammation, and volume overload (8). These factors are highly correlated with atrial electrical and structural remodeling, contributing to the promotion and perpetuation of AF. The atrial electrical remodeling refers to the changes in electrical dysfunction influenced by the ion channels, the disequilibrium of intracellular Ca^2+^ homeostasis and gap junctions (9), and atrial structural remodeling includes atrial enlargement, cardiomyocytes hypertrophy, and interstitial fibrosis (10). Much evidence indicates that the chronic intra-atrial or intra-ventricular volume overload and the activation of RAAS and SNS resulting from CKD could urge stress relative signaling pathways [e.g., tumor necrosis factor-α, transforming growth factor-β1 (TGF-β1), and NAPDH oxidases, etc.], leading to myocyte apoptosis, interstitial fibrosis and the alteration of electrical activity (8). Besides, the enhanced RAAS could act synergistically with oxidative stress and inflammatory regulators such as TGFβ1 and NLR (nucleotide-binding domain leucine-rich repeat-containing receptor) pyrin domain-containing protein 3 (NLRP3) inflammasome to promote atrial structural remodeling and AF eventually (11, 12). Atrial connexins play an important role in maintaining the homogeneity of atrial electrical conduction and are easy to be influenced by the activation of inflammation and oxidative stress and the atrial structural remodeling (11). Therefore, the occurrence and development of atrial fibrillation with CKD could be multifactorial and multi-targets and Figure 1 presents the mechanism pattern of AF occurrence in CKD.

**Figure 1:**
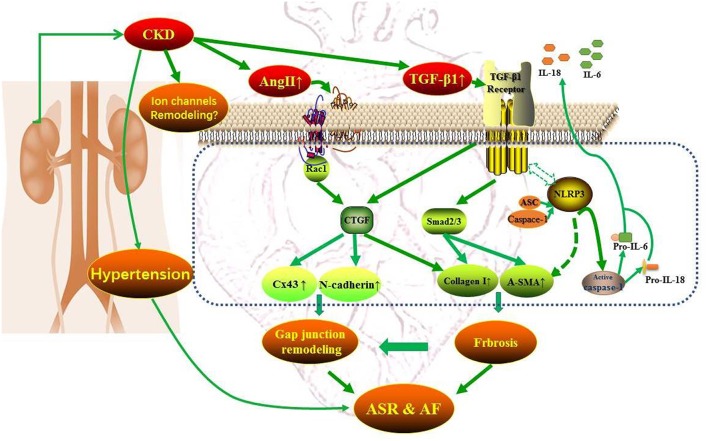
Mechanism pattern diagram of AF occurrence in CKD.

The coexistence of CKD and AF can present a challenge for treatment. Renal dysfunction increases the risk of bleeding and complications from anti-arrhythmic drugs of which are eliminated by kidney when patients are under anticoagulation and rate control (12, 13). Unfortunately, current (drug) therapies for AF are palliative due to the high rate of recurrence even excluding CKD (14). The strategies for AF therapies have been changing continuously from treatment to prevention—the upstream therapy for AF which is proposed to prevent AF development by inhibiting atrial remodeling including atrial structural and electrical remodeling (14, 15). For the existing difficulties in comprehensive treatment for both diseases, to prevent the prevalence of AF in CKD patients should be considered as one part of the upstream therapies for AF.

In the paper published in current issue of Frontiers, Qiu et al. showed that CKD resulted in left atrial enlargement, interstitial fibrosis, and increased AF inducibility, which may be related to the activation of TGF-β1/Smads and NLRP3 inflammasome signaling as well as connexins remodeling (16). These findings revealed that the pathologic features of CKD enhanced AF are multiplex and suggested several potential mediators of increased AF prevalence in CKD. The present study focused mainly on the atrial structural remodeling, including interstitial fibrosis and left atrial enlargement and the morphologic changes of electrocardiogram including P waves and PR interval. In combination with previous findings, the present study also identified CTGF and Rac-1 as the potential mediators of atrial structural remodeling (ASR). Most importantly, since the molecular mechanisms of the activations of TGF-β1/Smads, NLRP3 inflammasome, CTGF and Rac-1, and connexins remodeling all links to an increased angiotensin II, we can propose that a potential anti-AF approach for targeting the atrial structural remodeling by using angiotensin II antagonists is available to prevent AF in patients with CKD. Indeed, the main components of the RAAS, angiotensin II, is up-regulated in both AF patients and animals with CKD. However, the benefits of RAAS inhibition by angiotensin converting enzyme inhibitors or angiotensin receptor blockers with respect to the prevention of CKD induced AF have not been fully investigated. Therefore, the present study provides potential mediators involved in atrial structural remodeling, which may be constituted a promising option for the development of therapeutic strategy for AF upstream therapy.

However, the present study has several unfinished works and caveats. The features of atrial electrical remodeling in a CKD setting still remain unclear. The present study didn't mention any alterations related to the possible remodeling of ion channels, which is very likely to occur in this model. The findings of connexins remodeling and the morphological changes of P waves are far from adequate to elucidate the atrial electrical remodeling. Besides, it is important to investigate the functional changes of connexins beyond its alterations in protein expression and distribution and to ascertain whether the alterations of atrial connexins are cause or coincidence of AF in patients with CKD. Hypertension as a cause and consequence of CKD is also a strong risk factor for developing AF. In the present study, they didn't rule out the arrhythmogenic potential from hypertension. Additionally, a deeper interpretation of these molecular targets that contribute to atrial structural remodeling under CKD condition is needed. Regardless of these limitations, it is very encouraging to establish such an animal model and to investigate interdisciplinary research.

## Author Contributions

DZ and ZZ conceived and designed this research. LW, YF, and FL provided materials for writing this manuscript. DZ and LW wrote the manuscript.

### Conflict of Interest Statement

The authors declare that the research was conducted in the absence of any commercial or financial relationships that could be construed as a potential conflict of interest.

## References

[1] Herzog CA Asinger RW Berger AK Charytan DM Diez J Hart RG. Cardiovascular disease in chronic kidney disease. A clinical update from Kidney Disease: Improving Global Outcomes (KDIGO) Kidney Int. (2011) 80:572–86. 10.1038/ki.2011.223 21750584

[2] Mark PB. Strategies to manage cardiovascular risk in chronic kidney disease. Nephrol Dial Transplant. (2018) 33:23–5. 10.1093/ndt/gfx329 29237023

[3] Soliman EZ Prineas RJ Go AS Xie DW Lash JP Rahman M. Chronic kidney disease and prevalent atrial fibrillation: the Chronic Renal Insufficiency Cohort (CRIC). Am Heart J. (2010) 159:1102–7. 10.1016/j.ahj.2010.03.027 20569726 PMC2891979

[4] Alonso A Lopez FL Matsushita K Loehr LR Agarwal SK Chen LY. Chronic kidney disease is associated with the incidence of atrial fibrillation the Atherosclerosis Risk in Communities (ARIC) study. Circulation. (2011) 123:2946–81. 10.1161/CIRCULATIONAHA.111.020982 21646496 PMC3139978

[5] Nelson SE Shroff GR Li SL Herzog CA. Impact of chronic kidney disease on risk of incident atrial fibrillation and subsequent survival in medicare patients. J Am Heart Assoc. (2012) 1:1–10. 10.1161/JAHA.112.002097 23130165 PMC3487349

[6] Horio T Iwashima Y Kamide K Tokudome T Yoshihara F Nakamura S. Chronic kidney disease as an independent risk factor for new-onset atrial fibrillation in hypertensive patients. J Hypertens. (2010) 28:1738–44. 10.1097/HJH.0b013e32833a7dfe 20485194

[7] Navaravong L Barakat M Burgon N Mahnkopf C Koopmann M Ranjan R. Improvement in estimated glomerular filtration rate in patients with chronic kidney disease undergoing catheter ablation for atrial fibrillation. J Cardiovasc Electrophysiol. (2015) 26:21–7. 10.1111/jce.12530 25142836

[8] Voroneanu L Ortiz A Nistor I Covic A. Atrial fibrillation in chronic kidney disease. Eur J Intern Med. (2016) 33:3–13. 10.1016/j.ejim.2016.04.007 27155803

[9] Everett TH Olgin JE. Basic mechanisms of atrial fibrillation. Cardiol Clin. (2004) 22:9–20. 10.1016/S0733-8651(03)00117-6 14994844

[10] Everett TH Olgin JE. Atrial fibrosis and the mechanisms of atrial fibrillation. Heart Rhythm. (2007) 4:S24–7. 10.1016/j.hrthm.2006.12.040 17336879 PMC1850572

[11] Adam O Lavall D Theobald K Hohl M Grube M Ameling S. Rac1-induced connective tissue growth factor regulates connexin 43 and N-cadherin expression in atrial fibrillation. J Am Coll Cardiol. (2010) 55:469–80. 10.1016/j.jacc.2009.08.064 20117462

[12] Chen G Chelu MG Dobrev D Li N. Cardiomyocyte inflammasome signaling in cardiomyopathies and atrial fibrillation: mechanisms and potential therapeutic implications. Front Physiol. (2018) 9:1115. 10.3389/fphys.2018.01115 30150941 PMC6100656

[13] Lip GYH Frison L Grind M Invest S. Angiotensin converting enzyme inhibitor and angiotensin receptor blockade use in relation to outcomes in anticoagulated patients with atrial fibrillation. J Intern Med. (2007) 261:577–86. 10.1111/j.1365-2796.2007.01780.x 17547713

[14] Poli D Antonucci E Zanazzi M Grifoni E Testa S Ageno W. Impact of glomerular filtration estimate on bleeding risk in very old patients treated with vitamin K antagonists Results of EPICA study on the behalf of FCSA (Italian Federation of Anticoagulation Clinics). Thromb Haemost. (2012) 107:1100–6. 10.1160/TH11-10-0721 22535516

[15] Camm AJ Kirchhof P Lip GYH Schotten U Savelieva I Ernst S. Guidelines for the management of atrial fibrillation The Task Force for the Management of Atrial Fibrillation of the European Society of Cardiology (ESC). Europace. (2010) 12:1360–420. 10.1093/eurheartj/ehq278 20876603

[16] Qiu HL Ji CL Liu W Wu YC Lu ZY Lin QZ. Chronic kidney disease increases atrial fibrillation inducibility: involvement of inflammation, atrial fibrosis, and connexins. Front Physiol. (2018) 9:1726. 10.3389/fphys.2018.01726 30564139 PMC6288485

